# Supplementary material for: Evaluation of Blood Soluble CD26 as a Complementary Biomarker for Colorectal Cancer Screening Programs
Source: Cancers (Basel). 2022 Sep 20;14(19):4563. doi: 10.3390/cancers14194563 (PMC9559671; doi:10.3390/cancers14194563)
Supplement: Supplementary file 1 [file cancers-14-04563-s001.zip › Suppl Table S1.pdf]

Supplementary Table S1. Levels of serum sCD26 and DPP-IV activity according to colonoscopy findings among men.

**Pathology**	**sCD26** Mean±SD (ng/mL)			**DPP4** Mean±SD (mU/mL)			**Total protein** Mean ± SD (mg/mL)			**Sp. Act (DPP4/prot)** Mean ± SD (mU/mg)			**sCD26/DPP4 ratio** Mean ± SD (ng/mU)		
	N		*p*-value	N		*p*-value	N		*p*-value	N		*p*-value	N		*p*-value
**No neoplasia**	470	496.27±182.78	**<0.001**[1]	299	40.30±11.15	**<0.001**[1]	299	76.49±13.58	0.277[1]	299	0.60±1.02	**0.002**[1]	293	12.80±6.06	**0.022**[1]
NCF	112	533.42±199.74	-	51	42.15±13.73	-	51	73.51±10.06	-	51	0.93±2.43	-	49	13.95±5.07	-
hemorrhoids	41	527.93±174.64	0.877[2]	18	39.46±12.16	0.466[2]	18	76.66±9.57	0.252[2]	18	0.52±0.18	0.483[2]	17	11.46±5.32	0.090[2]
diverticula	35	416.29±155.16	**0.002**[2]	17	42.63±9.62	0.895[2]	17	71.54±9.50	0.482[2]	17	0.60±0.15	0.583[2]	17	10.46±3.22	**0.010**[2]
polyps*	25	454.86±152.28	0.067[2]	10	35.12±9.59	0.129[2]	10	76.30±8.02	0.412[2]	10	0.47±0.14	0.553[2]	9	12.10±2.92	0.295[2]
others†	12	514.23±120.59	0.745[2]	11	40.98±7.56	0.787[2]	11	79.33±12.88	0.103[2]	11	0.52±0.11	0.586[2]	11	13.30±5.58	0.705[2]
NAA	245	488.75±181.12	**0.037**[2]	343	39.91±10.65	0.211[2]	192	77.55±15.08	0.072[2]	192	0.53±0.17	**0.026**[2]	190	12.83±6.61	0.270[2]
**Advanced neoplasia**	356	367.94±179.38	**<0.001**[3]	302	32.25±12.50	**<0.001**[3]	302	76.50±14.74	0.996[3]	302	0.43±0.17	**0.004**[3]	281	11.55±4.52	**0.005**[3]
AA	218	409.78±178.64	**<0.001**[4]	168	36.15.±11.87	**<0.001**[4]	168	77.71±12.70	0.339[4]	168	0.47±0.16	0.111[4]	147	11.96±5.07	0.151[4]
CRC	138	301.86±160.13	**<0.001**[5]	134	27.36±11.55	**<0.001**[5]	134	74.97±16.87	0.359[5]	134	0.37±0.18	**0.011**[5]	134	11.09±3.79	**0.003**[5]

p-value: [1]ANOVA test for comparison of the 6 no neoplasia subgroups; [2]Student´s t test for comparison of NCF vs each of the NN subgroups; [3]Student´s t test for comparison of no neoplasia vs advanced neoplasia; [4]Student´s t test for comparison of no neoplasia vs AA; [5]Student´s t test for comparison of no neoplasia vs CRC; NCF: no colorectal findings; *inflammatory and hyperplasic polyps; †others include angiodysplasia, rectitis and melanosis coli among others; NAA: non-advanced adenomas; AA: advanced adenomas; CRC: colorectal cancer.
