# Supplementary material for: Evaluation of Blood Soluble CD26 as a Complementary Biomarker for Colorectal Cancer Screening Programs
Source: Cancers (Basel). 2022 Sep 20;14(19):4563. doi: 10.3390/cancers14194563 (PMC9559671; doi:10.3390/cancers14194563)
Supplement: Supplementary file 1 [file cancers-14-04563-s001.zip › Suppl Table S2.pdf]

Supplementary Table S2. Levels of serum sCD26 and DPP-IV activity according to colonoscopy findings among women.

Pathology	sCD26 Mean±SD (ng/mL)			DPP4 Mean±SD (mU/mL)			Total protein Mean ± SD (mg/mL)			Sp. Act (DPP4/prot) Mean ± SD (mU/mg)			sCD26/DPP4 ratio Mean ± SD (ng/mU)		
	N		*p*-value	N		*p*-value	N		*p*-value	N		*p*-value	N		*p*-value
**No neoplasia**	602	536.12±183.09	**<0.001**[1]	372	45.98±10.91	**<0.001**[1]	372	76.15±11.77	0.853[1]	372	0.62±0.17	**<0.001**[1]	368	11.97±4.14	0.685[1]
NCF	229	548.73±180.49	-	137	46.55±10.46	-	137	76.23±12.34	-	137	0.63±0.17	-	136	12.45±4.53	-
hemorrhoids	75	479.40±177.05	**0.004**[2]	37	43.69±12.87	0.162[2]	37	75.66±12.04	0.802[2]	37	0.59±0.16	0.123[2]	35	11.41±3.48	0.208[2]
diverticula	61	542.93±180.67	0.824[2]	35	43.05±9.77	0.076[2]	35	75.07±10.14	0.608[2]	35	0.58±0.13	0.108[2]	34	11.25±3.52	0.153[2]
polyps*	20	514.17±120.93	0.402[2]	4	42.31±5.35	0.422[2]	4	71.01±6.66	0.401[2]	4	0.60±0.13	0.800[2]	4	10.72±1.81	0.448[2]
others†	12	568.37±245.15	0.719[2]	8	56.70±13.59	**0.010**[2]	8	73.95±10.53	0.609[2]	8	0.79±0.28	0.133[2]	8	11.51±6.20	0.578[2]
NAA	205	541.03±187.79	0.664[2]	151	46.24±10.70	0.804[2]	151	76.70±11.77	0.745[2]	151	0.62±0.17	0.598[2]	151	11.89±3.96	0.261[2]
**Advanced neoplasia**	238	402.64±170.33	**<0.001**[3]	201	36.82±12.77	**<0.001**[3]	201	77.31±15.16	0.349[3]	201	0.48±0.17	**<0.001**[3]	195	11.45±7.14	0.276[3]
AA	127	437.41±150.26	**<0.001**[4]	93	41.69.10.06	**<0.001**[4]	93	78.26±10.72	0.117[4]	93	0.54±0.15	**<0.001**[4]	87	10.98±3.36	**0.019**[4]
CRC	111	362.86±183.44	**<0.001**[5]	108	32.63±13.41	**<0.001**[5]	108	76.48±18.15	0.857[5]	108	0.44±0.17	**<0.001**[3]	108	11.84±9.11	0.828[5]

p-value: [1]ANOVA test for comparison of the 6 no neoplasia subgroups; [2]Student´s t test for comparison of NCF vs each of the NN subgroups; [3]Student´s t test for comparison of no neoplasia vs advanced neoplasia; [4]Student´s t test for comparison of no neoplasia vs AA; [5]Student´s t test for comparison of no neoplasia vs CRC; NCF: no colorectal findings; *inflammatory and hyperplasic polyps; †others include angiodysplasia, rectitis and melanosis coli among others; NAA: non-advanced adenomas; AA: advanced adenomas; CRC: colorectal cancer.
